# A Genome for *Bidens hawaiensis*: A Member of a Hexaploid Hawaiian Plant Adaptive Radiation

**DOI:** 10.1093/jhered/esab077

**Published:** 2022-01-04

**Authors:** M Renee Bellinger, Erin M Datlof, Karen E Selph, Timothy J Gallaher, Matthew L Knope

**Affiliations:** 1 From the Department of Biology, University of Hawaiʻi at Hilo, 200 West Kāwili Street, Hilo, HI 96720, USA; 2 Department of Oceanography, University of Hawaiʻi at Mānoa, 1000 Pope Road, Honolulu, HI 96822, USA; 3 Department of Natural Sciences, Bishop Museum, 1525 Bernice Street, Honolulu, HI 96817, USA

**Keywords:** Asteraceae, flow cytometry, koʻokoʻolau, monoploid genomes, PacBio HiFi, polyploid

## Abstract

The plant genus *Bidens* (Asteraceae or Compositae; Coreopsidae) is a species-rich and circumglobally distributed taxon. The 19 hexaploid species endemic to the Hawaiian Islands are considered an iconic example of adaptive radiation, of which many are imperiled and of high conservation concern. Until now, no genomic resources were available for this genus, which may serve as a model system for understanding the evolutionary genomics of explosive plant diversification. Here, we present a high-quality reference genome for the Hawaiʻi Island endemic species *B. hawaiensis* A. Gray reconstructed from long-read, high-fidelity sequences generated on a Pacific Biosciences Sequel II System. The haplotype-aware, draft genome assembly consisted of ~6.67 Giga bases (Gb), close to the holoploid genome size estimate of 7.56 Gb (±0.44 SD) determined by flow cytometry. After removal of alternate haplotigs and contaminant filtering, the consensus haploid reference genome was comprised of 15 904 contigs containing ~3.48 Gb, with a contig N50 value of 422 594. The high interspersed repeat content of the genome, approximately 74%, along with hexaploid status, contributed to assembly fragmentation. Both the haplotype-aware and consensus haploid assemblies recovered >96% of Benchmarking Universal Single-Copy Orthologs. Yet, the removal of alternate haplotigs did not substantially reduce the proportion of duplicated benchmarking genes (~79% vs. ~68%). This reference genome will support future work on the speciation process during adaptive radiation, including resolving evolutionary relationships, determining the genomic basis of trait evolution, and supporting ongoing conservation efforts.

The biota of the Hawaiian Islands has long served as an evolutionary model for understanding the drivers of speciation and phenotypic diversification (e.g., Gulick 1872; Carlquist 1974; Gillespie et al. 2020). However, despite these and many other detailed assessments, elucidating the relationships between the underlying genome evolution and ecomorphological diversification processes that occur during speciation and adaptive radiation has been hindered by a lack of whole-genome resources. Although genomes from Hawaiian animal taxa that have undergone adaptive radiation have recently been sequenced (e.g., Callicrate et al. 2014; Kang et al. 2016), the lack of available reference genomes for native Hawaiian plant adaptive radiations hinders our ability to understand speciation processes and the development of plant genome–phenome relationships.

The striking diversity of Hawaiian angiosperms results largely from adaptive radiation, where a single colonist species often rapidly diversifies into many descendent species all adapted to different ecological niches (e.g., Schluter 2000). The native Hawaiian angiosperm flora is comprised of approximately 1020 plant species all descended from the long-distance dispersal of only 259 original colonists (Price and Wagner 2018). Some of these lineages, including the iconic Hawaiian silverswords, lobeliads, and *Bidens* (koʻokoʻolau), have undergone adaptive radiation producing high species richness and extreme ecomorphological divergence (Carr 1987; Baldwin and Sanderson 1998; Givnish et al. 2009; Knope et al. 2012). This limited number of founders relative to extant species richness is due to the extreme isolation of the islands (~3700 km for the nearest mainland source of propagules), which built de novo over millions of years as the Pacific tectonic plate slowly passed over a stationary “hot spot” in the Earth’s mantle where plumes of molten magma break through the seafloor (Wilson 1963; Price and Clague 2002). Across the 8 main Hawaiian islands, in situ evolution and adaptive diversification have been promoted by a combination of factors. These include (but are not limited to) geographic isolation, both among and within islands, highly heterogeneous landscapes often including multiple volcanoes on single islands, and extreme variation in climate and weather patterns associated with prevailing winds and island topography (Price and Wagner 2004).

The genus *Bidens* is circumglobally distributed, with ~150–230 species found across 5 continents (Kimball and Crawford 2004), and throughout Polynesia (Knope et al. 2020a). Colonization of the Hawaiian islands by *Bidens* is believed to have occurred as a single founding event by a hexaploid ancestor (Gillett and Lim 1970), followed by subsequent diversification into the currently recognized 19 species and 8 subspecies (Ganders and Nagata 1984; Knope et al. 2012). Crown group diversification for this group was estimated to have begun 1.32 Mya (0.66–2.10, 95% highest posterior density) (Knope et al. 2020a). Remarkably, the endemic Hawaiian *Bidens* exhibit greater morphological and ecological diversity than all continental members of the genus combined (Ganders and Nagata 1984; Helenurm and Ganders 1985). Yet, despite Hawaiian *Bidens*’ tremendous ecomorphological variation, their level of genetic differentiation among species is comparable to the level of genetic differentiation found among populations within single continental plant species, based on isozyme loci (Helenurm and Ganders 1985). In addition, they also display low levels of genetic differentiation within their plastomes and nuclear internal transcribed spacer regions (Knope et al. 2020b). This disparity between rates of genetic differentiation and rates of ecomorphological evolution highlights a fundamental question in evolutionary genetics: how do lineages diversify greatly in phenotype with little apparent genetic diversification?

The objective of this study was to generate a Hawaiian *Bidens* genome assembly to support investigations of the genomic basis of trait evolution, aid in phylogenetic and taxonomic studies, and, in turn, inform conservation efforts. We used long-read Pacific Biosciences (PacBio) high-fidelity (HiFi) sequencing data for genome sequencing and estimated the genome size using flow cytometry. A single-copy ortholog analysis was applied to compare attributes of the *Bidens* genome assembly to other Asteraceae (or Compositae) and select polyploid species. Given Hawaiian *Bidens* unusually high level of ecomorphological diversification, this newly available genome resource allows this clade to serve as a model system for understanding the evolutionary genomics of rapid plant diversification, especially for polyploid species.

## Methods

### Biological Materials

We collected a *B. hawaiensis* plant from the eastern side of Hawai‘i island (originating from Kalapana, Puna), subjected it to clonal propagation, and used leaves sourced from clones for high-molecular-weight (HMW) DNA extraction for genome sequencing and cell nuclei extraction for genome size estimation. A sterile specimen (Datlof 10, BISH 782333) was vouchered with the Bernice Pauahi Bishop Museum, Honolulu, Hawaii. A previously sampled *B. hawaiensis* plant that originated from Hawaiʻi Volcanoes National Park, on Hawaiʻi Island, was subjected to short-read sequencing as described in Knope et al. (2020b) and used here for assembly benchmarking.

### Genome Profiling

Knowledge of genome size is useful for determining the number of sequences needed to perform genome assembly and to determine how closely a consensus haploid genome assembly approximates the size of the haploid or (averaged) monoploid genomes. For clarity, haploid and monoploid genomes are respectively defined as: 1) the lowest recognized level of generative polyploidy in haplophase, where *n* = x, and 2) one chromosome set of an organism and its DNA having the chromosome base number x (Greilhuber et al. 2005). Although Hawaiian *Bidens* are known to be hexaploid (Gillett and Lim 1970) and possess a base number of 12 chromosomes (2*n* = 6x = 72; Ballard 1986), how their polyploidy evolved is not yet understood. The *B. hawaiensis* 1C-value (the DNA content of a single nonreplicated monoploid genome; Greilhuber et al. 2005) was unknown during the project planning stage, but we estimated it would range between 1 to 3 Giga bases (Gb), based on Kew database records for 9 other *Bidens* species (diploid and tetraploid only; no data available for hexaploids) (Leitch et al. 2019). Here, we measured the *B. hawaiensis* holoploid genome size (the DNA content of the whole chromosome complement; Greilhuber et al. 2005) with flow cytometry, using as a calibration standard the tetraploid tomato *Lycopersicon esculentum* “Gardener’s Delight,” having a 4C-value of 4 pg (Obermayer et al. 2002), where 1 pg DNA = 0.978 × 10^9^ bp (Doležel et al. 2007). The *L. esculentum* and *B. hawaiensis* nuclei were run individually (4 technical replicates from multiple leaves of a single *B. hawaiensis* plant; 4 biological replicates from 4 *L. esculentum* plants), and together (6 replicates total, with the single *B. hawaiensis* plant combined with 2 replicate pairs of 3 *L. esculentum* plants) to test for fluorescence shifts of DNA peaks that could occur due to secondary compound interference. The plant nuclei were extracted from freshly emerged leaf tissues by finely dicing ~2-cm^2^-sized portions of leaf into small pieces (<<1 mm) in the presence of nuclei extraction buffer (Sysmex) followed by propidium iodide staining with a Sysmex CyStain kit. Analyses were performed using a Beckman-Coulter CyoFLEX S flow cytometer with 561 nm excitation (30 mW) and 610 ± 20 nm emission to detect propidium iodide, collecting a minimum of 10 000 nuclei per determination. For each sample, a ratio of the mean positions of the haploid peaks of *L. esculentum* and *B. hawaiensis* was multiplied by the 2C DNA content of *L. esculentum* to obtain the unknown DNA content of *B. hawaiensis*. Genome size calculations followed the methodology described by Doležel et al. (2007).

Reference-free genome profiling based on k-mer spectrum analysis produces estimates of genome characteristics such as size, repetitiveness, and heterozygosity (Ranallo-Benavidez et al. 2020). We calculated k-mer frequencies of long- and short-read sequencing datasets with Jellyfish 2 (Marçais and Kingsford 2011) and modeled results with Genomescope v2 (Ranallo-Benavidez et al. 2020). The model fit was evaluated across a range of k-mer sizes (from 17 to 21), with the ploidy level set to 6 and coverage (kcov) settings estimated from an initial run using default values. The HiFi and HiSeq sequencing datasets were analyzed separately.

### Nucleic Acid Library Preparation

Freshly emerged leaves (about 40) were removed from plant clones using sterile technique, snap-frozen in a dry ice and ethanol bath within 2 min of excision from the stem, and transferred on dry ice to the Arizona Genomics Institute (AGI) sequencing facility at the University of Arizona for HMW DNA extraction using a modified CTAB protocol. The DNA extract size profile, visualized with pulse-field electrophoresis (BioRad CHEF), indicated the presence of full chromosome arms. After using a Covaris g-tube to fragment the DNA, a PacBio sequencing library was prepared and size-selected on a Sage BluePippen instrument with S1 marker, setting the size range to 10–25 kb. This yielded an average input library size of 16 kb. The DNA extraction protocol for the short-read dataset is available from Knope et al. (2020b).

### DNA Sequencing and Genome Assembly

Program versions and parameter settings are available from Table 1.

**Table 1.** A list of programs, versions, parameters, and datasets used to produce and select a *Bidens hawaiensis* reference genome assembly

Purpose	Software	Settings and associated programs	Data input or result
Reference-free genome profiling	GenomeScope2	K-mer = 17; kcov = 13	HiFi and HiSeq sequences
Genome assembly	Canu v2.0	correctedErrorRate = 0.015 batOptions = “-eg 0.01 -eM 0.01 -dg 6 -db 6 -dr 1 -ca 50 -cp 5” -pacbio-corrected	HiFi; **Asm1**
	Canu v2.0	correctedErrorRate = 0.015 batOptions = “-eg 0.01 -eM 0.01 -dg 6 -db 6 -dr 1 -ca 50 -cp 5” contigFilter = “2 0 1.0 0.5 0” -pacbio-corrected	HiFi; **Asm2**
	Canu v2.0, hi_canu fork	correctedErrorRate = 0.015 batOptions = “-eg 0.01 -eM 0.01 -dg 6 -db 6 -dr 1 -ca 50 -cp 5” pacbio-hifi	HiFi; **HiCanu**
Duplicate purge for haploid consensus	minimap2 for Purge_Dups	assembly-reference mapping parameter (-x) = asm20; self-self mapping parameter (-x) = asm5	All assemblies
	Purge_Dups	purge_dups calcuts -l 2 -m 9 -u 30	Asm1, Asm2
	Purge_Dups	purge_dups calcuts -l 2 -m 6 -u 27	HiCanu
Assembly metrics	QUAST v5.0.2	Default settings	All assemblies
Assembly completeness	bwa-MEM 0.7.17 SAMtools v1.9	Read mapping: bwa mem -M Read filtering: samtools view -bh -q 20 -f 3 -F 2316	All haploid consensus assemblies
	BUSCO 4.0.5	Augustus v3.2.3, Blast+ v2.2.31, HMMER v3.2,	All assemblies
		OrthoDB Obd10 eudicot database eudicots_odb10.2019-11-20	
Genome architecture	RepeatModeler 2.0	-q, custom repeat library	All haploid consensus assemblies
	RepeatScout v1.0.06	Default settings	‘’
	RECON v. 1.08	Default settings	‘’
	RepeatMasker version open-4-1-1	-s	‘’
	RMBlastN 2.10.0	Dfam v3.3 (download date 2020-11-09)	‘’
Organelle contig identification	MitoFinder v 1.4	--new-genes, arwen mtDNA: --max-contig-size 325 000 bp chloroplast: --max-contig-size 200 000 bp	HiCanu primary and alternate contigs
	mummer-4.0.0beta2	--maxmatch -l 100	‘’

Analyses included: reference-free genome profiling; draft genome assembly (Asm1, Asm2, and HiCanu, result indicated by bold) using Pacific Biosystems HiFi sequence data and 3 sets of assembly parameters; removal of alternate contigs to produce consensus haploid assemblies; assembly benchmarking; repetitive content analysis; and identification of organelle contigs (HiCanu assembly only).

#### DNA Sequencing

The AGI prepared size-selected HMW DNA for circular consensus sequencing (CCS) on a PacBio Sequel II (Wenger et al. 2019) platform. Two Single Molecule, Real-Time (SMRT) 8M cells were loaded at 70pM Overpressured-Layer Chromatography and run in CCS mode for 30 h. Those 2 cells produced 9.4 million raw sequences representing 850 Gb raw data, from which we obtained 3.83 million HiFi sequences having an average size of 15.1 kb and N50 length of 13.5 kb. The average read quality score of HiFi sequences was QV34, and the total number of high-quality (>Q20) bases was 51.7 billion.

The quality trimmed, short-read dataset used for assembly benchmarking consisted of ~13.9 million paired-end reads sequenced on an Illumina HiSeq 4000 using 150 cycles, for a total of ~4.2 Gb of data (National Center for Biotechnology Information [NCBI] SRA SRS5133635; Knope et al. 2020b).

#### Genome Assembly

Genome assembly was optimized by testing 3 sets of assembly parameters, post-processing each of the 3 draft, haplotype-aware genomes (labeled as Asm1, Asm2, and HiCanu) followed by separation of primary and alternate contigs to produce haploid consensus representations. Genome assembly was performed using the Canu assembly framework, applying standard settings recommended in Nurk et al. (2020), with some modifications. The Asm1 assembly was generated with standard settings only, while the Asm2 assembly used the same settings, with exception of disabling the low-coverage contig filter. The HiCanu assembly was generated using standard settings, and incorporated the experimental homopolymer compression function (hi_canu fork; -pacbio-hifi) to test whether that setting would increase haplotype separation (evidenced by a larger assembly) and provide better repeat resolution, with an expected cost of increased assembly fragmentation. Each genome assembly took 36–48 h to complete on a high performance computing core, using one node with 128 Gb RAM and 20 cores.

#### Post-assembly Processing

Most long-read genome assemblers construct a haplotype-fused mosaic representation of the diploid (or polyploid) genome that is post-assembly processed to produce a consensus haploid representation consisting of primary contigs (the haploid reference) and alternate contigs (Chin et al. 2016; Weisenfeld et al. 2017; Zhang et al. 2020). For diploids, an ideal consensus haploid representation consists of all homozygous and hemizygous regions from both haplomes as well as one allelic copy of all heterozygous regions in the 2 haplomes, such that any region in either haplome aligns to only a single location in the consensus haploid assembly (Roach et al. 2018). Genome assembly for polyploids is challenging because constituent monoploid genomes may be highly similar, depending on whether the whole-genome duplication (WGD) event occurred through processes of autoploidy or alloploidy, ongoing meiotic pairing and chiasma, and whether the WGD event was recent or ancient. Thus, for hexaploids, a haploid consensus assembly might consist of mosaic sequences that represent one to multiple sets of chromosomal pairs per component genome, A_1_A_2_A_3_ (autopolyploid), A_1_A_2_B_1_ (auto- and allopolyploid), or A_1_B_1_C_1_ (allopolyploid). As such, a haploid consensus might collapse to a range of single, double, or triploid monoploid genomes, A, AB, or ABC. We generated a haploid reference assembly by removing duplicate contigs (defined as repetitive, artifactual, and redundant haplotigs) from the initial draft assembly using program Purge_dups (Guan et al. 2020), which considers sequence similarity and read coverage depth and identifies up to 2 allelic sequences (pairwise duplicate blocks) during analysis. To accommodate the hexaploid status of *B. hawaiensis*, that program was run sequentially by successively passing the primary and alternate contigs output file as the input file for the next iteration until fewer than 10% of the total number of contigs were identified as duplicates. The Purge_dup (Guan et al. 2020) coverage cutoff settings were estimated from that program’s read-coverage histogram outputs. The highest quality haploid consensus assembly, identified from benchmarking results, was selected to serve as the reference genome.

The reference haploid consensus assembly and corresponding alternate contigs were filtered for contaminants. Based on the NCBI genome submission portal’s built-in contaminant screen, leading and trailing sections of contigs that contained overlooked internal adapter sequences were manually trimmed, and contigs identified as bacterial in origin were removed. Contigs that putatively originated from organelles were identified by MitoFinder (Allio et al. 2020) implemented with references *Helianthus annuus* (mitochondria, NCBI accession NC_023337.1) and *B. hawaiensis* (chloroplast, NCBI accession NC_047259; Knope et al. 2020b). To control for false assignments, contigs were considered nuclear in origin if they failed to align back to their respective organelle reference, with alignments performed using the MUMmer program nucmer (Kurtz et al. 2004), or if they contained fewer than 5 complete genes or had a maximum depth of coverage <100x. The putative organelle contigs were placed in a separate data repository (see Data Availability section).

#### Assembly Quality Evaluation

The quality of each haploid consensus assembly was evaluated using metrics of total numbers of assembled bases, contiguity, completeness, and short-read sequence mapping data. The genome contiguity was evaluated by the numbers of contigs and the N50 score, the contig length at which 50% of the total bases fall in a given assembly, as measured by QUAST (Gurevich et al. 2013). To quantitatively measure the completeness of genome assemblies, we applied the Benchmarking Universal Single-Copy Orthologs (BUSCOs) (Seppey et al. 2019) pipeline, which produces evolutionarily informed expectations of gene content from near-universal single-copy orthologs. That pipeline accessed programs Augustus (Stanke et al. 2008), Blast+ (Altschul et al. 1990), and HMMER (Eddy 1998), and utilized a OrthoDB Obd10 eudicot database containing 2326 single-copy genes from 31 species (Kriventseva et al. 2019). For comparative purposes, BUSCOs were also analyzed for genomes of 12 other Asteraceae (all genomes publicly available as of May 2020), and genomes of 5 polyploid eudicots (see Supplementary Table 1 for genome accessions and details). The quality of each haploid consensus assembly was further assessed using the ratio of the number of unique short reads to the total numbers of short reads mapped to each assembly using bwa-MEM (Li 2013) and filtered with SAMtools (Li et al. 2009).

#### Genome Architecture

The repetitive content of each *B. hawaiensis* consensus haploid genome assembly was characterized by generating de novo repeat libraries with RepeatModeler (Flynn et al. 2020), a de novo transposable element (TE) family identification and modeling package that finds interspersed repeats by integrating RepeatScout (Price et al. 2005) and RECON (Bao and Eddy 2002). The custom repeat library served to identify and quantify interspersed repeats, simple repeats, and low-complexity regions based on the slow (-s) search option of RepeatMasker (Smit et al. n.d.) and RMBlastN, first replacing the default (small) RepeatMasker Dfam database with an updated and more comprehensive version, v3.3 (download date 9 November 2020) (Hubley et al. 2016). The de novo repetitive content determined here was compared to results from the k-mer spectrum analysis.

## Results

### Genome Profiling

**Figure 1.**
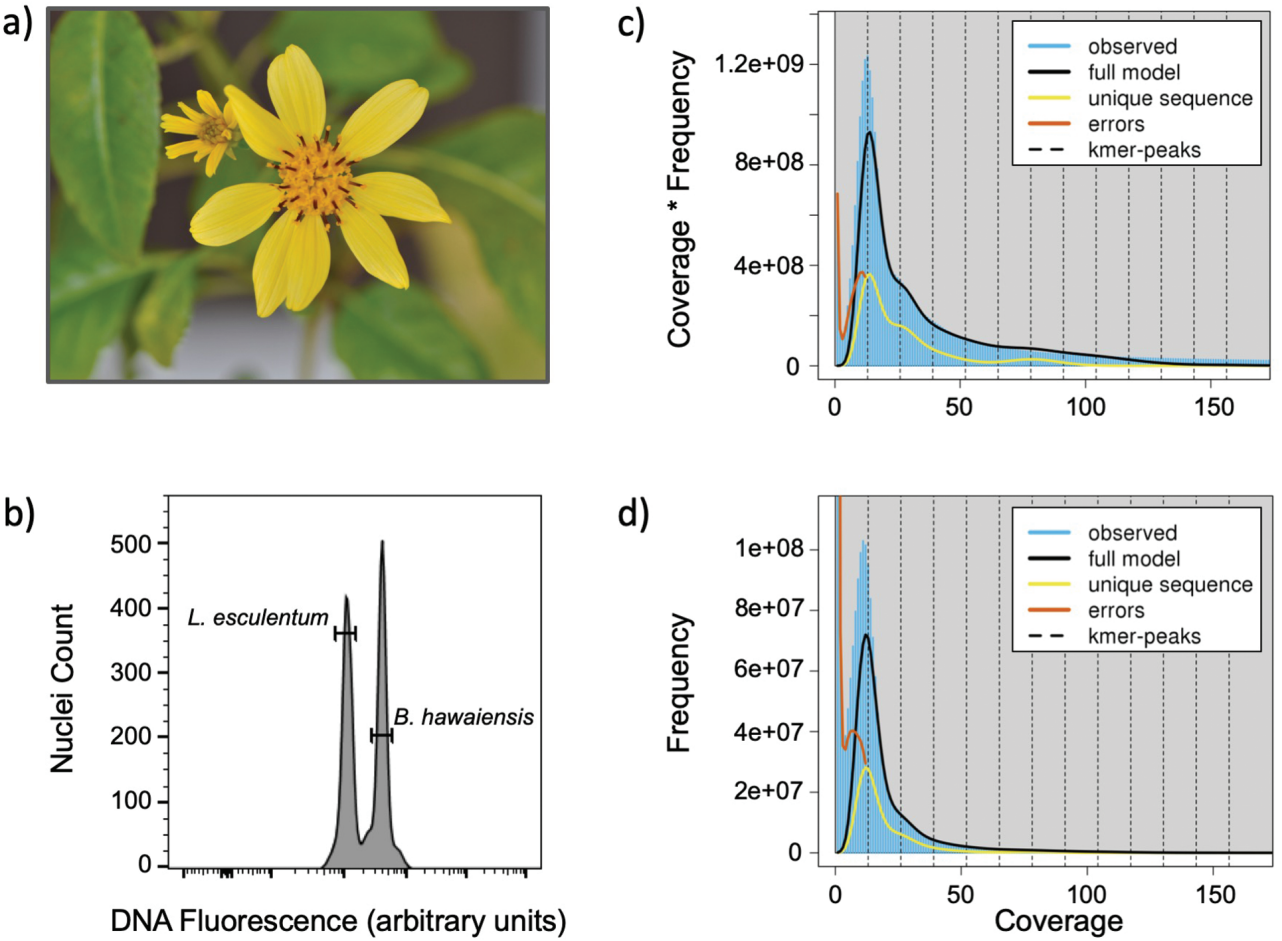
A profile of *Bidens hawaiensis*. (**a**) A *B. hawaiensis* flower photographed from the plant used for flow cytometry and genome sequencing. (**b**) An example flow cytometry data plot from one replicate of a sample with both *Lycopersicon esculentum* and *B. hawaiensis*. Shown are the number of nuclei as a function of the DNA fluorescence (arbitrary units) from propidium iodide staining. Data for the mean peak position of each replicate sample (*n* = 10) are available from Supplementary Table 2. (**c**, **d**) K-mer spectrum analysis of HiFi data. (c) The 3 monoploid genomes of *B. hawaiensis* can be detected as peaks in the coverage ∗ frequency plot. (d) A plot showing that a dominant k-mer frequency peak occurs at approximately 12x coverage, and that numerous k-mers with modest coverage were miscategorized as sequencing errors.

The flow cytometry estimate of the *B. hawaiensis* holoploid genome size was 7.56 Gb (±0.44 SD), based on the mean peak position of 10 replicate samples (Figure 1a and b; see Supplementary Table 2). The haploid genome size estimate based on k-mer spectrum analysis of HiFi sequence data was 495 Mb (see Supplementary Table 3), far smaller than 3C expectations (if all monoploid genomes were distinct, e.g., ABC) based on flow cytometry results, 3.78 Gb (±0.22 SD). Adjusting the GenomeScope2 model parameters k-mer size (17–21) and kcov (5–13) had little impact on model outputs. The best-fit GenomeScope2 model, generated with setting k-mer = 17 and kcov = 13, revealed k-mer coverage peaks for each of the 3 monoploid chromosome sets (Figure 1c) and found that the *B. hawaiensis* genome contains a repetitive content totaling 80% of the haploid genome size (see Supplementary Table 3). This high repeat content likely contributed to the smaller than expected k-mer-based genome size calculation, because k-mers that originate from repetitive regions are likely to be collapsed during the k-mer count process. Another factor contributing to the low k-mer-based genome size estimate is the modest proportion of sequences that were (mis)categorized as sequencing errors, which are most evident in the coverage ∗ frequency plot as visualized as a peak intermediate (and shifted left) to that of the full model (Figure 1d). A portion of sequences classified as errors clearly tracks the full model result and falls within the main observed data peak. The k-mer spectrum genome size estimate from short-read data, ~10 Mbp (data not shown), was even smaller than results based on HiFi sequence data. But given the short-read data were of low coverage, it is probable that the dataset was insufficient for genome size modeling. In sum, the k-mer spectrum analysis of both long and short reads produced unreasonably small genome size estimates and was unreliable for determining genome size and, by extension, assembly completeness.

### Genome Assembly

The 3 initial draft assemblies were similarly sized, ~6.4 Gb to ~6.7 Gb, but varied by numbers of contigs, ranging from ~52k to ~65k (Table 2). Post-assembly, a single duplicate-purge step resulted in haploid consensus assemblies that were about half the size of each assembly draft (43–52% fewer bases), and contained substantially fewer contigs (75–80% reduction). Additional rounds of duplicate purging were ineffective, with <1% difference in assembly sizes and further BUSCO losses; therefore, results from the second round of duplicate purging were discarded. The HiCanu haploid consensus assembly was selected as the reference genome because of its modestly higher numbers of concordantly mapped short reads and slightly higher recovery of complete of benchmarking genes (Table 2; see Supplementary Figure 1).

**Table 2.** Descriptive statistics for 3 *Bidens hawaiensis* draft genome assemblies (Draft) and associated haploid consensus (Haploid) assemblies produced by duplicate purging

Type	Assembly	Contigs	Total # assembled bases	N50	BUSCO score (S+D)	Total # mapped short reads	Concordantly mapped short reads
Draft	Asm1	52 363	6 354 280 161	304 617	96.7%	n/a	n/a
	Asm2	54 599	6 401 020 890	298 996	96.3%	n/a	n/a
	HiCanu	64 728	6 669 162 087	187 741	96.4%	n/a	n/a
Haploid	Asm1	10 514	2 748 720 504	707 276	95.6%	28 048 504 (99.3%)	16 684 169 (59.5%)
	Asm2	10 913	2 756 276 744	701 909	95.2%	28 049 029 (99.3%)	16 700 646 (59.5%)
	HiCanu	15 958	3 478 010 306	421 857	96.6%	27 967 161 (99.6%)	18 436 392 (65.9%)

Assembly quality metrics include numbers of contigs, assembly length, N50, BUSCO scores (single and duplicate genes, S+D), and for duplicate purged assemblies, the total numbers of mapped and concordantly mapped Illumina short reads.

#### Benchmarking Analysis

**Figure 2.**
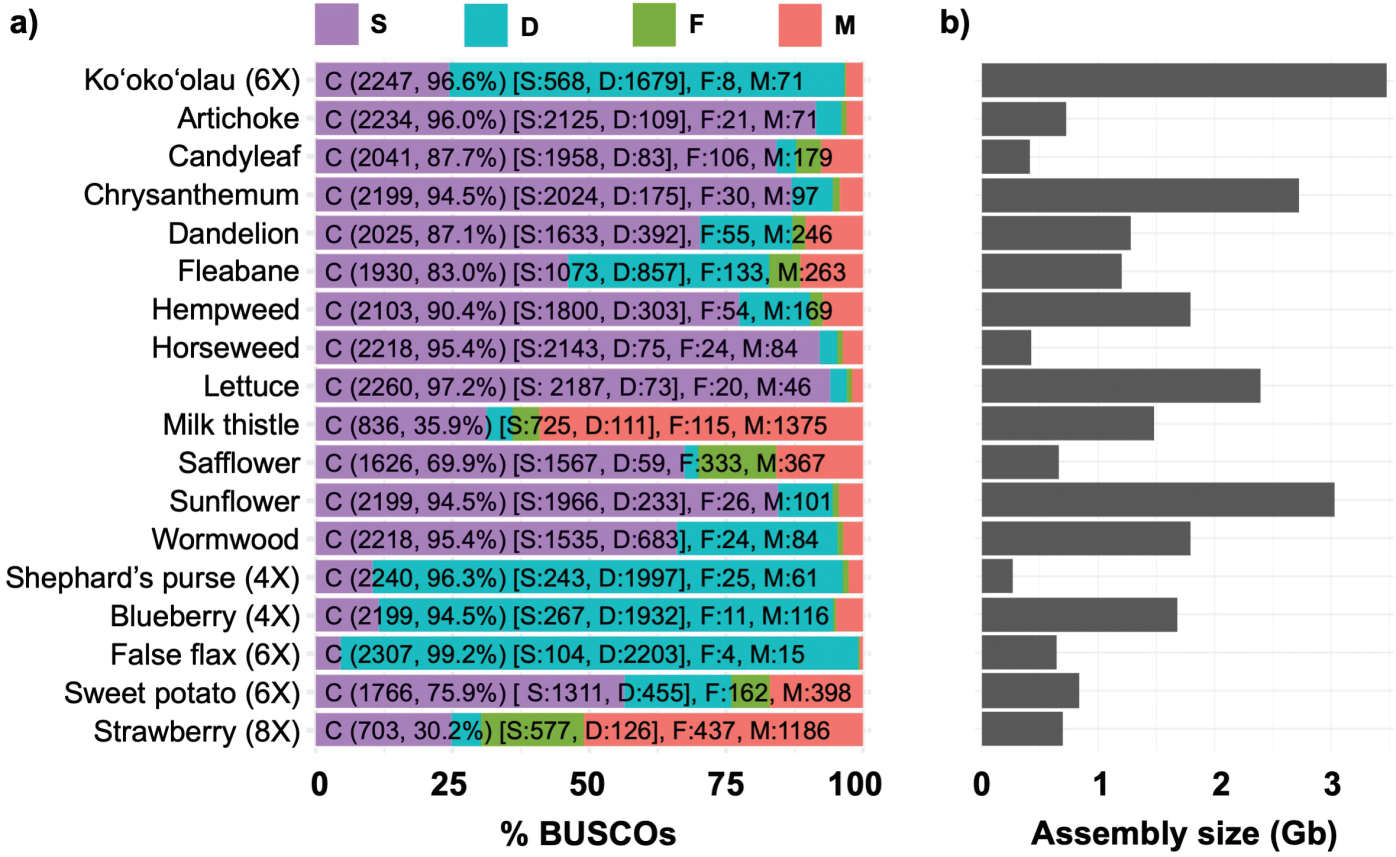
Comparative assessments of genome completeness and size. The genome assembly comparisons include the hexaploid Ko’oko’olau (*Bidens hawaiensis*; haploid consensus assembly), 12 diploid Asteraceae, and 5 polyploid eudicots (ploidy number in parenthesis). (**a**) Benchmarking Universal Single-Copy Ortholog (BUSCO) analysis with the eudicot ODB10 database containing 2326 genes. Shown are the proportion and numbers of BUSCO gene recoveries categorized as complete and single-copy (S), complete and duplicated (D), fragmented (F), or missing (M). (**b**) Total number of assembled bases for each genome. See Supplementary Table 1 for scientific names, genome accessions (when available), and genome references.

The BUSCO analyses indicates that the *B. hawaiensis* HiCanu consensus haploid assembly was highly complete, with recovery of >96.6% single-copy orthologs (Figure 2a). Compared to 12 other Asteraceae genome assemblies, this *Bidens* genome was the second most complete, despite its substantially larger assembly size (Figure 2a and b). Comparisons among the three *Bidens* draft assemblies shows that the assembly methodology had little impact on BUSCO scores, and the proportion of complete, duplicated BUSCOs remained high, *~*68% compared to ~79%, despite duplicate purging (see Supplementary Table 4 and Supplementary Figure 1). This overall high level of duplication was consistent with patterns observed across nearly all of the 5 other polyploid plant genome assemblies (Figure 2a).

#### Genome Architecture

Based on analysis with RepeatModeler and associated programs, the total interspersed repeat content of *B. hawaiensis* consensus haploid assemblies was estimated to range from 70% to 74% (see Supplementary Table 5). The HiCanu assembly methodology recovered the highest proportion of repetitive content, as expected, because that assembly methodology is designed to increase haplotype separation. Among the characterized retroelements, the majority were long terminal repeat (LTR) retrotransposons (at 34.5%). In contrast, DNA transposons were estimated to comprise <2% of the genome. The estimated proportion of genome repetitive content was surprisingly similar to the repetitive content estimated by the reference-free k-mer spectrum result, at 80%.

#### Reference Genome and Contaminant Filtering

The final HiCanu haploid consensus reference genome assembly contained 15 904 contigs with a total length of 3 475 153 191 assembled bases (N50 value of 422 594), after trimming segments that contained internal adapter sequences (*n* = 3, all <15 kb length), removal of bacterial contaminants (*n* = 1 contig), and removal of contigs (*n* = 54) that putatively originated from organelles. The alternate contig dataset contained few internal adapter sequences (*n* = 7, all <15 kb length) and no bacteria contaminants, but did contain a high number (*n* = 2580) of putative organelle sequences. That high number could be due to assembly fragmentation that resulted from the repetitive content or organelles, including inverted repeats (which also leads to mis-assembly), and the complex nature of plant mitochondrial genomes, which can occur in branched, linear forms and subgenome-sized circles, and are known to recombine frequently leading to genome isomerization (Wang et al. 2018; Wang and Lanfear 2019; Kozik et al. 2019; Wynn and Christensen 2019). Additionally, a subset of contigs could represent organelle mis-assignment and/or mis-assemblies that occurred because of nuclear DNA copies of chloroplast-like or mitochondrial genes, as evidenced by peaks and valleys in depth of coverage.

## Discussion

As far as we are aware, this study presents the first genome available for a species from a Hawaiian plant adaptive radiation. We anticipate this genomic resource will support future research efforts to shed light on genetic underpinnings to ecomorphological diversifications that occur during speciation and adaptive radiation, improve phylogenomic and biogeographic hypotheses, and aid in conservation efforts of the highly imperiled Hawaiian *Bidens* taxa. In addition, this study provides a framework for the genome assembly of other polyploid plant taxa, in Hawaiʻi and elsewhere.

Recent work has highlighted that rapid species diversification and the generation of evolutionary novelty can include hybridization and WGDs (e.g., Gillespie et al. 2020). In the case of hybridization, novel gene combinations can enhance phenotypic variability, and in turn, evolutionary fitness, as has been observed in African cichlids and other taxa (Marques et al. 2019). All Hawaiian *Bidens* species that have been tested are cross-compatible (Gillett 1972; Ganders and Nagata 1984; Knope et al. 2013), and hybrid swarms are not uncommon (Gillett and Lim 1970; Knope and Datlof, personal observation). Globally, *Bidens* are found in diploid, tetraploid, and hexaploid states (Ballard 1986; Huang and Kao 2015), and all of the Hawaiian and Marquesan *Bidens* species that have been investigated are hexaploid (Gillett and Lim 1970). Although WGD events are proposed to promote phenotypic diversification (Otto and Whitton 2000; Ren et al. 2018), an enigma observed for angiosperms is that a species-rich clade and a species-poor sister clade often share the same ancestral WGD event, demonstrating that other factors must be involved as determinants of diversification beyond genome duplication (Schranz et al. 2012; Robertson et al. 2017; Carretero-Paulet and Van de Peer 2020). Helenurm and Ganders (1985) proposed that a limited number of genes controlling key morphological characters in *Bidens* likely exert disproportionate effects on trait evolution. If that is the case, diversification within *Bidens* is predicted to correlate with divergence of key regulatory genes (Barrier et al. 2001). Whether hybridization or WGD is a dominant mechanism for generating phenotypic innovations in *Bidens* is an exciting avenue of research ripe for exploration.

Ploidy level also has ramifications for breeding systems, as they relate to conservation biology. For example, hexaploid members of the highly invasive *B. pilosa* complex are considered self-compatible (Ballard 1986; Sun and Ganders 1990), while the also highly invasive, but tetraploid, *B. alba* is generally considered self-incompatible (Ballard 1986; Huang and Kao 2015). Yet, Knope et al. (2013) confirmed prior findings by Grombone-Guaratini et al. (2004), that some *B. alba* populations can be self-compatible, and found that *B. pilosa* and *B. alba*, which are highly invasive throughout Hawaiʻi and other Pacific Islands, are cross compatible, but with low pollen fertility, presumably due to their ploidy differences. Further, Knope et al. (2013) demonstrated that neither *B. pilosa* nor *B. alba* are cross-compatible with the hexaploid native Hawaiian species, which are themselves fully cross-compatible. Clearly, the relationship between ploidy level, breeding system, and the potential for hybridization is key to an improved understanding of the genomics of cross-compatability, information that can inform conservation efforts.

According to the International Union for the Conservation of Nature (IUCN) Red List, the Hawaiian *Bidens* are highly imperiled. Of the 27 taxa (19 species and 8 subspecies) endemic to Hawaiʻi, 12 are considered vulnerable or worse, with *B. cosmoides*, *B. forbesii*, and *B. valida* listed as endangered, and *B. campylotheca pentamera*, *B. sandvicensis confusa*, and *B. wiebkei* listed as critically endangered with the highest risk of extinction in the wild (IUCN 2020). Conservation genomics can guide the primary objectives of breeding programs, conserving the highest possible level of genetic diversity to prevent inbreeding depression and support adaptive response in the face of changing environmental conditions (Hedrick and Miller 1992; Allendorf et al. 2010). Should ex situ conservation be implemented in a conservation plan for any of the Hawaiian *Bidens* species, this genome reference could be applied to detect cryptic hybrids, guide founder selection, and enable tools to measure and monitor genome-wide diversity.

This *B. hawaiensis* hexaploid genome assembly was constructed using highly accurate long reads produced by only 2 SMRT 8M cells at an estimated ~15x coverage per monoploid genome (1x = 3C = 36), which highlights the capacity of low-coverage HiFi sequencing to produce high-quality genomes, even for polyploid species. Long-read sequences are essential for maximizing genome quality because, compared to short reads, they are capable of resolving complex repeats that can otherwise lead to genome mis-assembly or fragmentation (Rhie et al. 2021). Still, de novo assembly of genomes remains a critical unsolved technical challenge, because genome assemblers typically focus on the vast majority of bases that are invariant across homologous chromosomes, leading to assembly fragmentation in heterozygous subregions (Yang et al. 2017). Polyploids are especially heterozygous, because each haploid set of gametes contains multiple copies of the monoploid genome (Yang et al. 2017). In this study, the levels of heterozygosity and divergence among constituent monoploid genomes were sufficient for assembling a near-complete (but fragmented) genome, evidenced by the total number of assembled bases, at ~6.67 Gb, relative to the flow cytometry-based genome size estimate, at ~7.56 Gb. However, during the haploid consensus step, we were unable to fully purge alternate alleles from the draft assembly without losing single-copy ortholog benchmarking genes, and the overall proportion of duplicated single-copy orthologs in the haploid consensus assembly (~3.48 Gb) remained high. The process of separating haplotype-fused contigs to produce a consensus, single haploid representation is an active area of computational biology research (Koren et al. 2017; Cheng et al. 2021; Rhie et al. 2021). Future efforts to improve this *Bidens* genome assembly could include incorporating chromatin interactions to connect chromosome structure to genomic sequence, as is done using Hi-C (Belton et al. 2012). Alternatively, or in addition, a high-coverage dataset (>33x per monoploid genome) and alternative assembly strategy such as HiFiasm can preserve the contiguity of all haplotypes (Cheng et al. 2021) to produce a full haplotig-resolved genome assembly.

## Conclusions

The hexaploid *Bidens* reference genome described in this study is intended to serve as a foundation for advancing hypotheses related to the capacity of plants to undergo rapid ecomorphological diversification and has implications for conservation planning. This genome resource may be used as a reference to understand how evolutionary processes shape genomic diversity within Asteraceae, and to identify genomic regions that underpin formation of adaptive traits. Further, we demonstrate that low-coverage, long-read PacBio HiFi sequences can produce high-quality genome assembles, even for polyploid plants with large, repetitive genomes.

## Supplementary Material

esab077_suppl_Supplementary_Figure

esab077_suppl_Supplementary_Tables

## Data Availability

We have deposited the primary data underlying these analyses as follows: short-read and genome data for BioSample SAMN18676211 have been archived under the NCBI BioProjects PRJNA720684 and PRJNA722028. The PacBio HiFi CCS sequences are available from the short-read archive under SRR14191093. The primary contigs of the HiCanu haploid consensus reference genome assembly and alternate contigs are archived under GenBank accessions JAIQDT000000000 and JAIQDU000000000. The HiCanu assembly repetitive content library and organelles sequences (primary and alternate haplotigs) were deposited in Dryad under accessions doi:10.5061/dryad.0zpc86703.
