# Supplementary material for: A Genome for Bidens hawaiensis: A Member of a Hexaploid Hawaiian Plant Adaptive Radiation
Source: J Hered. 2022 Jan 4;113(2):205–14. doi: 10.1093/jhered/esab077 (PMC9113482; doi:10.1093/jhered/esab077)

**Supplementary Figure 1.** Percent BUSCO single-copy gene orthologs recovered from three *Bidens hawaiensis* draft haplotype-aware genome assemblies and corresponding duplicate purged, consensus haploid assemblies (denoted by _p). A total of 2326 single copy ortholog benchmarking genes were contained in the benchmarking database, eudicots_odb10.


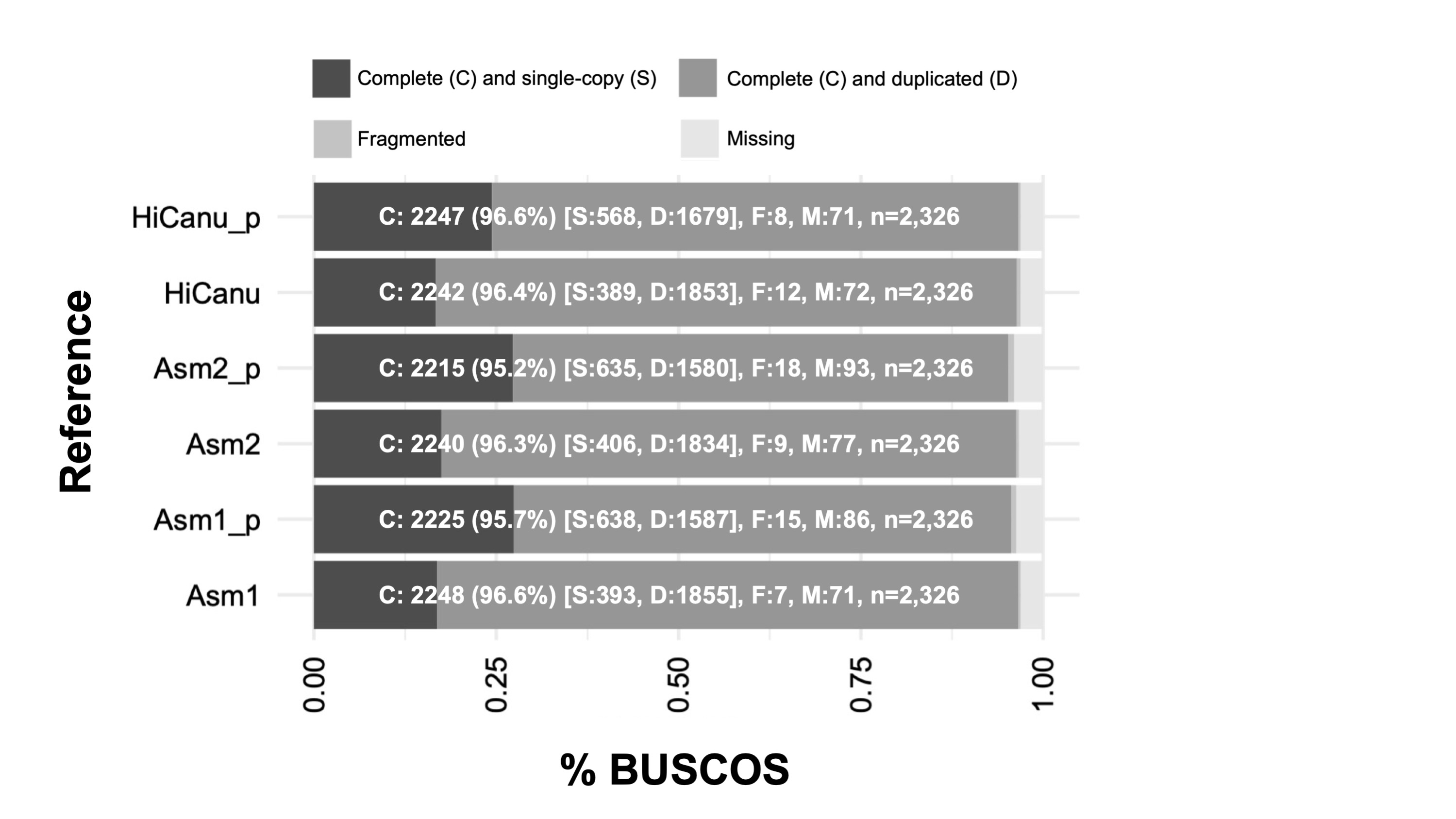

Supplement: esab077_suppl_Supplementary_Figure [file esab077_suppl_supplementary_figure.docx]
