# Supplementary material for: A Genome for Bidens hawaiensis: A Member of a Hexaploid Hawaiian Plant Adaptive Radiation
Source: J Hered. 2022 Jan 4;113(2):205–14. doi: 10.1093/jhered/esab077 (PMC9113482; doi:10.1093/jhered/esab077)
Supplement: esab077_suppl_Supplementary_Tables [file esab077_suppl_supplementary_tables.docx]

**Supplementary Table 1.** Characteristics of genomes used for comparative analysis of genome size and recovery of benchmarking universal single-copy orthologs (BUSCOs). Asteraceae (Compositae) are indicated by **bold** font. Abbreviations: Continuous Circular Reads = CCS; Continuous Long Reads = CLR; Illumina = I; Pacific Biosciences = PB; Roche 454 = 454; Sequence = Seq. Assembly information is from the USA’s National Center for Biotechnology Information GenBank repository.

| Species | Common Name | Sequencing Technology | Ploidy | Est. Genome Coverage | Total Sequence Length (with gaps) | # Scaffolds or (contigs) | | Scaffold (or contig) N50 | | Accession | | Reference |
| --- | --- | --- | --- | --- | --- | --- | --- | --- | --- | --- | --- | --- |
| ***Bidens hawaiensis*** | Koʻokoʻolau | PB Sequel II, CCS | Hexaploid | 14x | 3,236,624,620 | (14,005) | (473,555) | | JAIQDT000000000 | | This study | |
| ***Camelina sativa*** | False flax | I HiSeq; 454; BAC Clones; Genetic map | Hexaploid | 100x | 641,356,059 | 37,212 (72,248) | 30,099,736 (32,728) | | GCA_000633955.1 | | Kagale et al. 2014 | |
| ***Conyza (Erigeron) canadensis*** | Horseweed | PB Sequel, I; Hi-C | Diploid | 248x | 426,382,650 | 357 | 45,539,152 | | GCA_010389155.1 | | Laforest *et al.* 2020 | |
| ***Helianthus annuus*** | Sunflower | PB | Diploid | 100x | 3,027,844,889 | 1528 | 178,899,001 | | GCA_002127325.1 | | Badouin *et al.* 2017 | |
| ***Cynara cardunculus var. scolymus*** | Artichoke | HiSeq | Diploid | 80x | 725,197,765 | 13,588 | 125,941 | | GCA_001531365.1 | | Scaglione *et al.* 2016 | |
| ***Lactuca sativa*** | Lettuce | HiSeq | Diploid | 72x | 2,391,062,152 | 11,452 | 1,769,135 | | GCA_002870075.2 | | Reyes-Chin-Wo *et al.* 2017 | |
| ***Mikania micrantha*** | Climbing hempweed | PB RSII; PB Sequel | Diploid | 134x | 1,790,643,622 | 2,815 | 86,674,320 | | GCA_009363875.1 | | Liu *et al.* 2020 | |
| ***Artemisia annua*** | Sweet wormwood | 454; PB; MiSeq | Diploid | 180x | 1,792,856,094 | 39,400 | 104,891 | | GCA_003112345.1 | | Shen *et al.* 2018 | |
| ***Chrysanthemum seticuspe*** | Chrysanthemum | HiSeq; MiSeq | Diploid | 96x | 2,721,839,164 | 354,212 | 44,741 | | GCA_004359105.1 | | Hirakawa *et al.* 2019 | |
| ***Stevia rebaudiana*** | Candyleaf | HiSeq | Not reported | 116x | 411,383,069 | 55,557 | 37,276,437 | | GCA_009936405.1 | | Pirro and O’Neill 2020 | |
| ***Carthamus tinctorius*** | Safflower | HiSeq | Diploid | 21x | 661,937,708 | 463,906 | 3,565 | | GCA_001633085.1 | | Bowers *et al.* 2016 | |
| ***Silybum marianum*** | Milk thistle | HiSeq; PacBio | Diploid | 96x | 1,477,574,977 | (258,575) | (6967) | | GCA_001541825.1 | | ----- | |
| ***Erigeron breviscapus*** | Fleabane | PacBio RSII, CLR; I HiSeq | Diploid | 222x | 1,200,000,000 | 462,622 | 31,461 | | ^1^ | | Yang, Zhang, *et al.* 2017 | |
| ***Taraxacum kok-saghyz*** | Dandelion | PacBio RSII, CLR | Diploid | 48x | 1,277,495,208 | (31,965) | (47631) | | GWHAAAA00000000 | | Lin *et al.* 2018 | |
| *Ipomoea batatas* | Sweet potato | HiSeq | Hexaploid | 67x | 837,013,208 | 28,461 | 41,463,214 | | GCA_002525835.2 | | Yang, Moeinzadeh, *et al.* 2017 | |
| *Fragaria x ananassa* | Cultivated strawberry | I GAIIx; HiSeq | Octoploid | 399x | 697,762,269 | 625,966 | 2,201 | | GCA_000511835.1 | | Hirakawa *et al.* 2014 | |
| *Capsella bursa-pastoris* | Shepherd's purse | HiSeq, MiSeq | Allotetraploid | 40x | 268,430,517 | 8,186 | 627,605 | | GCA_001974645.1 | | Kasianov *et al.* 2017 | |
| *Vaccinium coymbosum* | Blueberry | 10x Genomics/I, HiC | Tetraploid | 324x | 1,679,081,592 | 48 | n/a | | PRJNA494180 | | Colle *et al.* 2019 | |

^1^ <http://gigadb.org/dataset/view/id/100290>

Badouin, H., Gouzy, J., Grassa, C.J., Murat, F., Staton, S.E., Cottret, L., Lelandais-Brière, C., Owens, G.L., Carrère, S., Mayjonade, B., Legrand, L., Gill, N., Kane, N.C., Bowers, J.E., Hubner, S., Bellec, A., Bérard, A., Bergès, H., Blanchet, N., Boniface, M.C., Brunel, D., Catrice, O., Chaidir, N., Claudel, C., Donnadieu, C., Faraut, T., Fievet, G., Helmstetter, N., King, M., Knapp, S.J., Lai, Z., Le Paslier, M.C., Lippi, Y., Lorenzon, L., Mandel, J.R., Marage, G., Marchand, G., Marquand, E., Bret-Mestries, E., Morien, E., Nambeesan, S., Nguyen, T., Pegot-Espagnet, P., Pouilly, N., Raftis, F., Sallet, E., Schiex, T., Thomas, J., Vandecasteele, C., Varès, D., Vear, F., Vautrin, S., Crespi, M., Mangin, B., Burke, J.M., Salse, J., Muños, S., Vincourt, P., Rieseberg, L.H., and Langlade, N.B., 2017. The sunflower genome provides insights into oil metabolism, flowering and Asterid evolution. *Nature*, 546, 148–152.

Bowers, J.E., Pearl, S.A., and Burke, J.M., 2016. Genetic Mapping of Millions of SNPs in Safflower (*Carthamus tinctorius* L.) via Whole-Genome Resequencing. *G3: Genes, Genomes, Genetics*, 6 (7), 2203–2211.

Colle, M., Leisner, C.P., Wai, C.M., Ou, S., Bird, K.A., Wang, J., Wisecaver, J.H., Yocca, A.E., Alger, E.I., Tang, H., Xiong, Z., Callow, P., Ben-Zvi, G., Brodt, A., Baruch, K., Swale, T., Shiue, L., Song, G.Q., Childs, K.L., Schilmiller, A., Vorsa, N., Robin Buell, C., Vanburen, R., Jiang, N., and Edger, P.P., 2019. Haplotype-phased genome and evolution of phytonutrient pathways of tetraploid blueberry. *GigaScience*, 8 (3), giz012.

Hirakawa, H., Shirasawa, K., Kosugi, S., Tashiro, K., Nakayama, S., Yamada, M., Kohara, M., Watanabe, A., Kishida, Y., Fujishiro, T., Tsuruoka, H., Minami, C., Sasamoto, S., Kato, M., Nanri, K., Komaki, A., Yanagi, T., Guoxin, Q., Maeda, F., Ishikawa, M., Kuhara, S., Sato, S., Tabata, S., and Isobe, S.N., 2014. Dissection of the octoploid strawberry genome by deep sequencing of the genomes of fragaria species. *DNA Research*, 21 (2), 169–181.

Hirakawa, H., Sumitomo, K., Hisamatsu, T., Nagano, S., Shirasawa, K., Higuchi, Y., Kusaba, M., Koshioka, M., Nakano, Y., Yagi, M., Yamaguchi, H., Taniguchi, K., Nakano, M., and Isobe, S.N., 2019. De novo whole-genome assembly in *Chrysanthemum seticuspe*, a model species of Chrysanthemums, and its application to genetic and gene discovery analysis. *DNA Research*, 26 (3), 195–203.

Kagale S,. Koh C., Nixon J., Bollina V., Clarke W.E., Tuteja R., Spillane C., Robinson S.J., Links M.G., Clarke C., Higgins E.E., 2014. The emerging biofuel crop *Camelina sativa* retains a highly undifferentiated hexaploid genome structure. *Nature communications*, 5 (1), 1-11.

Kasianov, A.S., Klepikova, A. V., Kulakovskiy, I. V., Gerasimov, E.S., Fedotova, A. V., Besedina, E.G., Kondrashov, A.S., Logacheva, M.D., and Penin, A.A., 2017. High-quality genome assembly of *Capsella bursa-pastoris* reveals asymmetry of regulatory elements at early stages of polyploid genome evolution. *Plant Journal*, 91, 278–291.

Laforest, M., Martin, S.L., Bisaillon, K., Soufiane, B., Meloche, S., and Page, E., 2020. A chromosome-scale draft sequence of the Canada fleabane genome. *Pest Management Science*, 76, 2158–2169.

Lin, T., Xu, X., Ruan, J., Liu, S., Wu, S., Shao, X., Wang, X., Gan, L., Qin, B., Yang, Y., Cheng, Z., Yang, S., Zhang, Z., Xiong, G., Huang, S., Yu, H., and Li, J., 2018. Genome analysis of *Taraxacum kok-saghyz* Rodin provides new insights into rubber biosynthesis. *National Science Review*, 5 (1), 78-87.

Liu, B., Yan, J., Li, W., Yin, L., Li, P., Yu, H., Xing, L., Cai, M., Wang, H., Zhao, M., Zheng, J., Sun, F., Wang, Z., Jiang, Z., Ou, Q., Li, S., Qu, L., Zhang, Q., Zheng, Y., Qiao, X., Xi, Y., Zhang, Y., Jiang, F., Huang, C., Liu, C., Ren, Y., Wang, S., Liu, H., Guo, J., Wang, H., Dong, H., Peng, C., Qian, W., Fan, W., and Wan, F., 2020. *Mikania micrantha* genome provides insights into the molecular mechanism of rapid growth. *Nature Communications*, 11, 340.

Pirro, S. and O’Neill, K., 2020. The complete genome sequence of Stevia rebaudiana, the Sweetleaf. *F1000Research*, 9, 751.

Reyes-Chin-Wo, S., Wang, Z., Yang, X., Kozik, A., Arikit, S., Song, C., Xia, L., Froenicke, L., Lavelle, D.O., Truco, M.J., Xia, R., Zhu, S., Xu, C., Xu, H., Xu, X., Cox, K., Korf, I., Meyers, B.C., and Michelmore, R.W., 2017. Genome assembly with in vitro proximity ligation data and whole-genome triplication in lettuce. *Nature Communications*, 8 (1), 1–11.

Scaglione, D., Reyes-Chin-Wo, S., Acquadro, A., Froenicke, L., Portis, E., Beitel, C., Tirone, M., Mauro, R., Lo Monaco, A., Mauromicale, G., Faccioli, P., Cattivelli, L., Rieseberg, L., Michelmore, R., and Lanteri, S., 2016. The genome sequence of the outbreeding globe artichoke constructed de novo incorporating a phase-aware low-pass sequencing strategy of F 1 progeny. *Scientific Reports*, 6 (1), 1–17.

Shen, Q., Zhang, L., Liao, Z., Wang, S., Yan, T., Shi, P., Liu, M., Fu, X., Pan, Q., Wang, Y., Lv, Z., Lu, X., Zhang, F., Jiang, W., Ma, Y., Chen, M., Hao, X., Li, L., Tang, Y., Lv, G., Zhou, Y., Sun, X., Brodelius, P.E., Rose, J.K.C., and Tang, K., 2018. The Genome of *Artemisia annua* Provides Insight into the Evolution of Asteraceae Family and Artemisinin Biosynthesis. *Molecular Plant*, 11, 776–788.

Yang, J., Moeinzadeh, M.H., Kuhl, H., Helmuth, J., Xiao, P., Haas, S., Liu, G., Zheng, J., Sun, Z., Fan, W., Deng, G., Wang, H., Hu, F., Zhao, S., Fernie, A.R., Boerno, S., Timmermann, B., Zhang, P., and Vingron, M., 2017. Haplotype-resolved sweet potato genome traces back its hexaploidization history. *Nature Plants*, 3, 696–703.

Yang, J., Zhang, G., Zhang, J., Liu, H., Chen, W., Wang, X., Li, Y., Dong, Y., and Yang, S., 2017. Hybrid de novo genome assembly of the Chinese herbal fleabane *Erigeron breviscapus*. *GigaScience*, 6, 1–7.

**Supplementary Table 2.** Leaf nuclei peak positions (mean ± standard deviation, arbitrary units) of *Lycopersicon esculentum* and *Bidens hawaiensis* from flow cytometry analyses of propidium iodide stained samples. The number of nuclei for each mean peak position is shown. The DNA content of *B. hawaiensis* is based on comparison to known DNA content of the internal standard *L. esculentum* of 2 pg (2C), and is shown for each replicate. The mean DNA content (± standard deviation) for *B. hawaiensis* of all 10 replicates is 7.73 ± 0.45 pg DNA.

| **Rep. No.** | ***L. esuclentum*** | | ***B. hawaiensis*** | | **DNA (pg)** |
| --- | --- | --- | --- | --- | --- |
|  | **No. Nuclei** | **Peak Position** | **No. Nuclei** | **Peak Position** |  |
| 1 | 12,273 | 91,154 ± 14,266 | 10,026 | 351,697 ± 47,526 | 7.72 |
| 2 | 46,858 | 72,658 ± 10,850 | 14,400 | 272,686 ± 42,368 | 7.51 |
| 3 | 10,024 | 96,252 ± 12,553 | 20,870 | 350,692 ± 43,047 | 7.29 |
| 4 | 20,354 | 136,250 ± 14,001 | 10,020 | 507,788 ± 45,100 | 7.45 |
| 5 | 18,115 | 102,864 ± 14,320 | 10,133 | 369,049 ± 41,268 | 7.18 |
| 6 | 12,398 | 97,748 ± 11,288 | 10,756 | 368,638 ± 35,840 | 7.54 |
| 7 | 27,231 | 89,554 ± 15,573 | 7,028 | 373,791 ± 35,308 | 8.35 |
| 8 | 14,782 | 67,864 ± 12,854 | 10,115 | 291,645 ± 49,491 | 8.59 |
| 9 | 13,482 | 64,594 ± 11,581 | 11,064 | 254,454 ± 40,639 | 7.88 |
| 10 | 15,926 | 58,171 ± 10,708 | 11,073 | 226,095 ± 35,005 | 7.77 |

**Supplementary Table 3.** Reference-free genome profiling using k-mer spectrum analysis. Data inputs include 3.83 million Pacific Biosciences High Fidelity (HiFi) sequences, with an average length of 15.1 Kb and totaling 55 Gb. The k-mer length and coverages were set to 17 and 13, respectively.

| Genome property | min | max |
| --- | --- | --- |
| Homozygous (aaaaaa) | 86.67% | n/a |
| Heterozygous (not aaaaaa) | 13.33% | n/a |
| Genome Haploid Length | 474,238,152 bp | 495,312,438 bp |
| Genome Repeat Length | 379,526,966 bp | 396,392,459 bp |
| Genome Unique Length | 94,711,186 bp | 98,919,980 bp |
| Model Fit | 48.64% | 87.14% |
| Read Error Rate | 0.53% | 0.53% |

**Supplementary Table 4.** Benchmarking universal single-copy orthologs (BUSCOs) recovered from three *Bidens hawaiensis* genome assemblies and their deduplicated, consensus haploid assembly representations. A total of 2326 single copy orthologs were contained in the benchmarking database eudicots_odb10. Gene recovery categories D and S refer to complete and duplicated (D) or complete and single (S).

|  | **Draft genome assembly** | | | | | | **Haploid consensus assembly** | | | | | |
| --- | --- | --- | --- | --- | --- | --- | --- | --- | --- | --- | --- | --- |
|  | **Numbers of genes** | | | **% BUSCOS** | | | **Numbers of genes** | | | **% BUSCOS** | | |
| BUSCOs | Asm1 | Asm2 | HiCanu | Asm1 | Asm2 | HiCanu | Asm1 | Asm2 | HiCanu | Asm1 | Asm2 | HiCanu |
| Total Complete | 2248 | 2240 | 2242 | 96.6% | 96.3% | 96.4% | 2215 | 2215 | 2247 | 95.2% | 95.2% | 96.6% |
| Complete + S | 393 | 406 | 389 | 16.9% | 17.5% | 16.7% | 638 | 635 | 568 | 27.4% | 27.3% | 24.4% |
| Complete + D | 1855 | 1834 | 1853 | 79.8% | 78.8% | 79.7% | 1587 | 1580 | 1679 | 68.2% | 67.9% | 72.2% |
| Fragmented | 7 | 9 | 12 | 0.3% | 0.4% | 0.5% | 15 | 18 | 8 | 0.6% | 0.8% | 0.3% |
| Missing | 71 | 77 | 72 | 3.1% | 3.3% | 3.1% | 86 | 93 | 71 | 3.7% | 4.0% | 3.1% |

**Supplementary Table 5.** Repetitive content of three consensus haploid assemblies of *Bidens hawaiensis* assembled from High-fidelity long-read sequences. See text for details.

|  | **Asm1** | | | **Asm2** | | | **HiCanu** | | |
| --- | --- | --- | --- | --- | --- | --- | --- | --- | --- |
| Category | Number of elements | Length occupied | % of sequence | Number of elements | Length  occupied | % of sequence | Number of elements | Length occupied | % of sequence |
| Retroelements | 533,695 | 858,214,586 | 31.22 | 511,060 | 827,837,290 | 30.03 | 812,733 | 1,232,071,106 | 35.42 |
| SINEs: | 0 | 0 | 0 | 0 | 0 | 0 | 0 | 0 | 0 |
| Penelope | 0 | 0 | 0 | 0 | 0 | 0 | 0 | 0 | 0 |
| LINEs: | 62,596 | 26,036,964 | 0.95 | 55,041 | 27,262,042 | 0.99 | 79,091 | 29,092,275 | 0.84 |
| CRE/SLACS | 33,727 | 11,463,941 | 0.42 | 24,941 | 10,222,325 | 0.37 | 34,795 | 11,630,122 | 0.33 |
| L2/CR1/Rex | 0 | 0 | 0 | 0 | 0 | 0 | 0 | 0 | 0 |
| R1/LOA/Jockey | 927 | 125,894 | 0 | 0 | 0 | 0 | 0 | 0 | 0 |
| R2/R4/NeSL | 0 | 0 | 0 | 0 | 0 | 0 | 0 | 0 | 0 |
| RTE/Bov-B | 10,105 | 3,961,442 | 0.14 | 10,036 | 6,647,315 | 0.24 | 23,031 | 5,032,803 | 0.14 |
| L1/CIN4 | 17,837 | 10,485,687 | 0.38 | 20,064 | 10,392,402 | 0.38 | 20,919 | 12,360,548 | 0.36 |
| LTR elements: | 471,099 | 832,177,622 | 30.28 | 456,019 | 800,575,248 | 29.05 | 733,642 | 1,202,978,831 | 34.59 |
| BEL/Pao | 117 | 62,862 | 0 | 0 | 0 | 0 | 0 | 0 | 0 |
| Ty1/Copia | 194,766 | 329,167,200 | 11.98 | 191,103 | 297,659,840 | 10.8 | 288,709 | 509,274,564 | 14.64 |
| Gypsy/DIRS1 | 272,431 | 498,994,497 | 18.15 | 262,571 | 499,892,415 | 18.14 | 439,967 | 688,363,136 | 19.79 |
| Retroviral | 0 | 0 | 0 | 0 | 0 | 0 | 0 | 0 | 0 |
|  |  |  |  |  |  |  |  |  |  |
| DNA transposons | 91,272 | 44,858,870 | 1.63 | 81,760 | 44,665,786 | 1.62 | 74,651 | 44,332,842 | 1.27 |
| hobo-Activator | 34,002 | 16,598,909 | 0.60 | 30,754 | 18,271,121 | 0.66 | 24,981 | 15,379,027 | 0.44 |
| Tc1-IS630-Pogo | 919 | 309,968 | 0.01 | 2,545 | 653,471 | 0.02 | 1,010 | 375,591 | 0.01 |
| En-Spm | 0 | 0 | 0 | 0 | 0 | 0 | 0 | 0 | 0 |
| MuDR-IS905 | 0 | 0 | 0 | 0 | 0 | 0 | 0 | 0 | 0 |
| PiggyBac | 0 | 0 | 0 | 0 | 0 | 0 | 0 | 0 | 0 |
| Tourist/Harbinger | 23,238 | 11,139,463 | 0.41 | 19,469 | 10,808,720 | 0.39 | 19,727 | 11,123,834 | 0.32 |
| Other (Mirage, | 0 | 0 | 0 | 0 | 0 | 0 | 0 | 0 | 0 |
| P-element, Transib) | |  |  |  |  |  |  |  |  |
| Rolling-circles | 19,615 | 9,524,157 | 0.35 | 31,846 | 13,553,046 | 0.49 | 26,754 | 11,637,235 | 0.33 |
| Unclassified: | 2,901,984 | 1,051,229,361 | 38.24 | 3,047,610 | 1,065,634,926 | 38.66 | 3,414,925 | 1,292,957,292 | 37.18 |
| Total interspersed repeats | | 1,954,302,817 | 71.1 |  | 1,938,138,002 | 70.32 |  | 2,569,361,240 | 73.87 |
|  |  |  |  |  |  |  |  |  |  |
| Simple repeats: | 1,363,118 | 66,885,067 | 2.43 | 1,366,474 | 67,045,618 | 2.43 | 1,674,772 | 82,274,142 | 2.37 |
| Low complexity: | 227,573 | 11,818,150 | 0.43 | 228,024 | 11,841,263 | 0.43 | 276,448 | 14,319,979 | 0.41 |
| **Total bases masked**: |  | 2,033,006,034 | 73.9 | n/a | 2,017,024,883 | 73.2 | n/a | 2,665,955,361 | 76.7 |
